# 

**Published:** 1882-09

**Authors:** 


### TABLE OF CONTENTS FOR SEPTEMBER, 1882.

### ORIGINAL COMMUNICATIONS.

PAGE.

ART. I.—Bacteria the Cause of Caries. By F. Y. CLARK, M. D., D.D.S.,.. 529

ORIGINAL TRANSLATIONS AND ABSTRACTS.

Early Joint Affections in Syphilis........ 537
Dyptheritic Cystitis........ 538
Surgery and the Doctrine of Evolution........ 539
The Treatment of Intussusception........ 540

SELECTIONS.

Otalgia from Reflex Dental Irritation........ 541
Columbus Medical College........ 544
Slaughter House Examinations........ 545
The Antiseptic Treatment of Typhoid Fever........ 546
The Health of London........ 546
The Smoke Nuisance........ 546
Chloroforming Mice........ 547

EDITORIAL........ 548

CURRENT NEWS AND OPINION........ 550

BIBLIOGRAPHICAL........ 552

POPULAR SCIENCE DEPARTMENT........ 554

